# Rosiridin Protects Against Aluminum Chloride-Induced Memory Impairment via Modulation of BDNF/NFκB/PI3K/Akt Pathway in Rats

**DOI:** 10.3390/medicina60111812

**Published:** 2024-11-04

**Authors:** Sana Saeed Alqarni, Muhammad Afzal, Khalid Saad Alharbi, Sattam Khulaif Alenezi, Tariq G. Alsahli, Shafqat Zaidi, Ahmed Essam Altyar, Nehmat Ghaboura, Imran Kazmi, Mohammad Jaffar Sadiq Mantargi, Faisal Imam

**Affiliations:** 1 Department of Clinical Laboratory Sciences, College of Applied Medical Science, King Saud University, Riyadh 11451, Saudi Arabia; saalqarni@ksu.edu.sa; 2 Pharmacy Program, Department of Pharmaceutical Sciences, Batterjee Medical College, Jeddah 21442, Saudi Arabia; jaffars909@gmail.com; 3 Department of Pharmacology and Toxicology, College of Pharmacy, Qassim University, Buraydah, Al-Qassim 51452, Saudi Arabia; khalid.alharbi9@qu.edu.sa (K.S.A.); sk.alenezi@qu.edu.sa (S.K.A.); 4 Department of Pharmacology, College of Pharmacy, Jouf University, Sakaka 72341, Saudi Arabia; tgalsahli@ju.edu.sa; 5 Department of Pharmacology, Teerthankar Mahaveer College of Pharmacy, Teerthankar Mahaveer University, Delhi Road, NH-24, Bagadpur, Moradabad 244001, India; zaidishafqat@gmail.com; 6 Department of Pharmacy Practice, Faculty of Pharmacy, King Abdulaziz University, Jeddah 21589, Saudi Arabia; aealtyar@kau.edu.sa; 7 Pharmacy Program, Department of Pharmacy Practice, Batterjee Medical College, Jeddah 21442, Saudi Arabia; pharmacy8.jed@bmc.edu.sa; 8 Department of Biochemistry, Faculty of Science, King Abdulaziz University, Jeddah 21589, Saudi Arabia; ikazmi@kau.edu.sa; 9 Department of Pharmacology and Toxicology, College of Pharmacy, King Saud University, Riyadh 11451, Saudi Arabia; fimam@ksu.edu.sa

**Keywords:** aluminum chloride, Alzheimer’s disease, brain-derived neurotrophic factor, cognitive impairment

## Abstract

*Background and Objectives:* Rosiridin is a monoterpene with outstanding monoamine inhibitory activity that is useful to treat depressive episodes and rapid-onset dementia. The current investigation aims to evaluate the neurologically protective impact of rosiridin, which opposes aluminum chloride (AlCl_3_) and causes memory dysfunction in rats. *Materials and Methods:* Memory impairment was developed in Wistar rats by administering AlCl_3_ (100 mg/kg p.o.) orally for 42 days and then supplemented with rosiridin at 10 and 20 mg/kg/p.o. Upon completion of the investigation, the behavior factor was performed utilizing the Y-maze, Morris Water Maze, and open field tests. Estimating numerous biological factors, such as nitric oxide (NO), oxidative stress (malondialdehyde MDA), acetylcholinesterase (AChE), butyrylcholinesterase levels (BuChE), antioxidants (glutathione GSH, catalase CAT, and superoxide dismutases SODs) and neurotransmitter (serotonin-5HT, dopamine-DA, acetylcholine-Ach) in the brain. Furthermore, interleukin-6 (IL-6), IL-1β, tumor necrosis factor (TNF-α), brain-derived neurotrophic factor (BNDF), nuclear factor kappa B (NFᴋB), phosphatidylinositol 3-kinase (PI3K), and pAkt were assessed in the diffused brain cells. *Results:* The rosiridin-treated group significantly improved in terms of behavioral parameters, including in the Y-maze, Morris Water Maze, and open field tests. Further, rosiridin restored biochemical parameters, including NO, oxidative stress AChE, BuChE, antioxidants, neurotransmitters, IL-6, IL-1β, TNF-α, BNDF, NFᴋB, PI3K, and pAkt compared to AlCl_3_. *Conclusions:* The current investigation reveals that rosiridin could ameliorate the impairment of memory that AlCl3 causes in rats via improvements in behavioral and restored biochemical parameters.

## 1. Introduction

Learning and memory are fundamental cognitive processes essential for the development of higher cognitive abilities [1]. Cognitive impairment is associated with neurodegenerative diseases, which become more prevalent with aging [2] and pose a growing threat to global public health [3]. Alzheimer’s disease (AD), characterized by memory loss, is driven by increased amyloid deposition, cholinergic network alterations, phosphorylated tau protein accumulation, and chronic dementia [2]. Despite a steady rise in the number of people affected by memory loss, the condition remains debilitating, often becoming a significant social and economic burden [4]. Current treatments include N-methyl-D-aspartate receptor antagonists and cholinesterase inhibitors aimed at reducing or slowing AD symptoms [5,6]. Allied cognitive impairment is a prevalent approach in learning and memory research, often interfering with cerebral signal transmission by blocking muscarinic acetylcholine (Ach) receptors, leading to neurocognitive issues [1,7].

Various behavioral animal models are used to study cognitive traits and dysfunctions [8]. Aluminum (Al) exposure, through food, water, air, and products used in agriculture and medicine, is linked to neurological damage in the hippocampus and cerebral cortex, further worsening cholinergic terminal deterioration in these regions [9,10]. Its gradual accumulation in the human body due to occupational or environmental factors can cause organ toxicity [11].

Aluminum chloride (AlCl_3_) can cross the blood–brain barrier (BBB), disrupting neuronal function and synaptic clefts, making the brain a primary target for Al toxicity [12]. Laboratory mice exposed to AlCl_3_ may develop cognitive and intellectual abnormalities, parasympathetic degeneration, amyloid plaques, and neurofibrillary tangles, any of which are pathogenic characteristics of AD [13]. Thus, AlCl_3_ has been used in this study to model AD-like cognitive impairment. Chronic aluminum exposure has been found to impair mitochondrial function in different brain regions, leading to increased free radical production and eventual DNA damage [14,15].

Various compounds, including those targeting the cholinergic system, synthetic anti-inflammatory and antioxidant agents, and herbal medicines, have demonstrated neuroprotective effects [16,17,18]. Essential oils contain monoterpenes, a large class of secondary plant metabolites with hydrocarbon structures. Monoterpenes and their derivatives play a significant role in the discovery and development of new bioactive compounds [19]. Monoterpenes have been found to exhibit anti-diabetic [20,21], cardioprotective [19,22,23], anti-inflammatory [19,24], antioxidant [25], anti-hyperlipidemic [26], anti-cancer [19,27], antimicrobial [19,28], antiviral [19,27], immunomodulatory [19,27], and antispasmodic properties. Recent interest in monoterpenes is due to their potential to prevent age-related neurodegeneration and modulate neuronal activity [29]. Dietary or plant-derived compounds rich in monoterpenes have been shown to enhance cognitive function in both humans and animals, suggesting they protect vulnerable neurons, support existing neuronal function, and promote neuroregeneration [30]. In neurodegenerative diseases, monoterpenes can influence gene expression, reduce oxidative stress, increase antioxidant enzyme activity, and decrease inflammatory mediators [31]. Numerous monoterpenes have shown neuroprotective potential in various forms of neurodegenerative disorders [31,32]. Rosiridin, a monoterpene found in *Rhodiola rosea* (*R. rosea*), exhibits strong monoamine inhibitory activity and has been used for rapid-onset treatment of depression and dementia [33,34,35]. In northeast Asian traditional medicine, the root of Rhodiola species (Crassulaceae) is utilized as an antiasthmatic, bleeding treatment, and antiaging therapy [36,37]. Previous studies have shown that Rhodiola root preparations positively impact cognitive function and act as antioxidants [38]. Previous research has demonstrated rosiridin’s positive effects against memory deficits induced by 3-nitropropionic acid, scopolamine, and streptozotocin-induced diabetes in rats [39,40,41]. However, no study has been conducted on how rosiridin affects cognitive impairment brought on by AlCl_3_. Therefore, this research aims to look at rosiridin’s neuroprotective potential towards AlCl_3_-induced cognitive decline in rats.

## 2. Methodology

### 2.1. Chemicals

Rosiridin (>98% purity, MSW Pharma, M.S., Chandrapur, India) was used in the study. IL-6, IL-1β, TNF-α, BNDF, NFᴋB, PI3K, and pAkt were analyzed by a rat ELISA kit procured from MSW Pharma, M.S., India.

### 2.2. Experimental Animals

Male Wistar adult rats weighing 180 ± 20 g (10–12 weeks old) were housed in regular propylene barracks with unlimited water and pellet food availability. A typical day–night cycle was maintained, and the rats were housed in groups at the usual room temperature (22 ± 2 °C). Protocol permission from the institutional research board of Batterjee Medical College, Jeddah, Saudi Arabia (RES-2023-0110), was obtained before any animal testing was conducted.

### 2.3. Study Plan

After being exposed to the outside environment for a minimum of seven days, a total of twenty-four animals were distributed at unknowns to four distinct categories (6 rats/group).

Groups:

- Control—each rat received a regular diet without any treatment;
- AlCl_3_—received AlCl_3_ (100 mg/kg p.o.) for 42 days [42,43];
- AlCl_3_ (100 mg/kg) + rosiridin (10 mg/kg p.o.);
- AlCl_3_ (100 mg/kg) + rosiridin (20 mg/kg p.o.).

**Figure 1:**
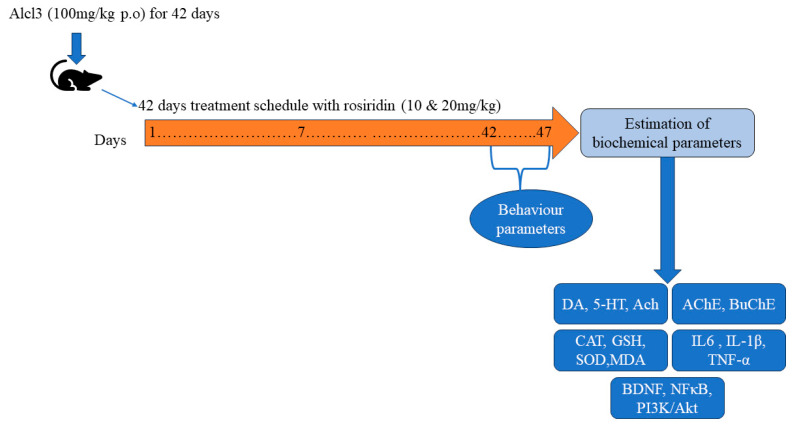
Experimental Design.

Freshly prepared rosiridin in a dimethyl sulfoxide (DMSO) dose was given every day for 42 days, with AlCl_3_ solution in distilled water given one hour prior. A behavioral test was conducted between days 42 and 47. Observing ethical guidelines, the animals were put to death on the forty-seventh day of the study following a twelve-hour fast by extracting the hippocampus which was homogenized for biological examination (Figure 1).

### 2.4. Parameters (Behavioral)

#### 2.4.1. Y-Maze Test

Spatial working memory was examined with the Y-maze test, which recorded random rearrangements. There were three arms (45 × 12 × 35 cm), each staggered by a 120° incline, in the wooden maze. In order to distinguish each arm, the walls were adorned with various designs and named A, B, and C. The rats were placed at the ends of each branch of the maze for free exploration. Each arm’s visits were counted for 5 min. For odor mitigation, the device was cleaned with 10% ethanol after each exercise. As a random alternation, three consecutive entries in three separate arms, like ABC, BCA, and CAB, were considered sequential [44].

#### 2.4.2. Morris Water Maze (MWM) Test

As stated earlier, the MWM assessment was utilized to assess rodent spatial learning [45,46,47]. The MWM round tank was divided into four equal quadrants or zones. For the first four days, an escape platform was positioned 1 cm below the water surface in one of the quadrants. Small white materials were scattered on the water’s surface. Each day during the learning phase (three trials per session), an animal was placed in the tank at a randomly selected starting point. The experiment commenced as soon as the animal was introduced to the tank and was stopped once the animal found and climbed onto the platform, with the average escape latency recorded. The maximum trial duration was 60 s. If the animal failed to reach the platform within 60 s, it was gently guided to the platform, and an escape latency of 60 s was recorded. The animal was allowed to remain on the platform for 20 s between trials. Immediately after completing all three trials, the animals were carefully dried and returned to their cages.

On the 5th day, a “probe trial” was conducted for 60 s to evaluate the rats’ memory of the hidden platform’s location. During this trial, the platform was removed from the tank, and the latency to locate the previous platform’s quadrant, as well as the time spent in that quadrant, were recorded [48].

#### 2.4.3. Open Field Test

One hour after the probe session, the rats were moved to an open field test device measuring 60 × 40 × 28 cm, with a floor divided into 12 squares. In the open field, rats were placed in the center of the apparatus for one minute. By recording the number of squares crossed in 5 min, the locomotive activity was examined [49].

### 2.5. Biochemical Analysis

#### 2.5.1. AChE and BuChE

Investigations were conducted on AChE and BuChE’s activities in brain tissue. First, a potter homogenizer running at 1200 rpm was used to create a 10% (*w*/*v*) brain homogenate in 0.9% standard saltwater. The homogenate had been centrifuged at 3000× *g* for 10 min at 4 °C to extract the supernatant. The supernatant was filtered and examined using the AChE and BuChE testing kits (MSW Pharma, M.S., India). AChE and BuChE activities were determined by analyzing Sym-Trinitrobenzene (TNB), a yellow-colored composite stated as U/mg of protein. Duplicate measurements were made for all samples [50].

#### 2.5.2. Estimation of MDA, GSH, SOD, and CAT

The Wills et al., approach was used for estimating the MDA level. The MDA content was expressed as nmol of MDA/mg of protein [51]. Ellman evaluated GSH with a method that has been formally documented [52]. The Misra and Frodvich approach were utilized to evaluate SOD [49,50,51,53]. The CAT function was ascertained by employing the technique outlined by Afzal et al. [39].

#### 2.5.3. Determination of Neurotransmitters

We utilized high-performance liquid chromatography (HPLC Agilent 1100 VWD Detector) to measure concentrations of various neurotransmitters, including serotonin (5 HT), dopamine (DA), and acetylcholine (Ach). ChemStation software version 10.02 was employed for data control and analysis [54].

#### 2.5.4. Nitric Oxide (NO)

Applying the described methodology, NO was ascertained [55]. In this study, the 5% 1-naphthylamine was replaced with 0.1% *w*/*v* naphthyl ethylene diamine dihydrochloride to modify the Griess reagent. The reagent solution (3 mL) was cultivated for 15 min at 25 °C with brain homogenates (2 mL) and phosphate-buffer saline (0.5 mL). The subsequent actions followed the same protocol as the preceding research. A pink chromophore was produced in dispersed light, and its absorbance was determined at 540 nm to the corresponding blank solutions. The NO concentration was shown as nmol/mg of tissue [56].

#### 2.5.5. Determination of Inflammatory Mediators

The levels of inflammatory mediators like TNF-α, IL-1β, and IL-6 were quantified using an ELISA kit. The concentration of these indicators was determined with calibration curves and reported in pg/mL. ELISA kits are assays performed on microwell plates pre-coated with antibodies [57].

#### 2.5.6. Estimation of BDNF, NFκB, and PI3K/pAkt

Employing the appropriate assay kits, ELISA kits measured BDNF, NFκB, and PI3K/pAkt levels in both control and treated animals. The samples were then added to the ELISA plate wells pre-coated with antibodies specific for each target protein (BDNF, NFκB, or PI3K/pAkt) [58].

### 2.6. Statistical Assessment

ANOVA was initially conducted to assess the presence of significant differences among the groups statistically. Numerical variables were tested using Shapiro–Wilk tests. With the exception of the MWM test, which was evaluated with two-way ANOVA and Bonferroni’s test, the findings of the subsequent variables were examined by one-way ANOVA and Tukey’s comparison test. The data are presented using GraphPad Prism software (Version 8.0.2) as mean ± SEM. *p* < 0.05 was chosen as the data relevance criterion.

## 3. Results

### 3.1. Behavioral Parameters

#### 3.1.1. Y-Maze Test

**Figure 2:**
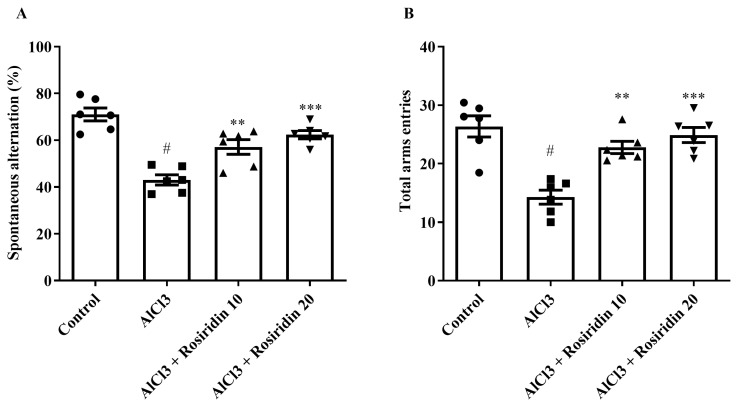
(**A**,**B**): Effects of rosirdin on Y-maze test. (**A**) Spontaneous alteration (%) and (**B**) total arm entries by using one-way ANOVA followed by Tukey’s post hoc test, respectively. Mean ± S.E.M. (*n* = 6). # *p* < 0.0001 vs. control; ** *p* < 0.001, *** *p* < 0.0001 vs. AlCl_3_-treated group.

The Y-maze test is used to measure spatial learning and memory. Compared to the control group, AlCl_3_ showed reduced spontaneous alteration and total arm entries. Rosiridin at both doses (10 and 20 mg/kg) significantly enhanced the spontaneous alteration [F (3, 20) = 21.66, (*p* < 0.0001)] and total arm entries [F (3, 20) = 15.60, (*p* < 0.0001)] compared to AlCl_3_-treated rats. The Y-maze test results are shown in Figure 2A,B. These findings suggest that rosiridin significantly improved cognitive function related to spatial learning and memory in AlCl_3_-treated rats.

#### 3.1.2. MWM Test

AlCl_3_ was shown to hinder the attention span of the rats in the MWM test, as evidenced by their faster response times when they stood on the fixed surface and departed from swimming. The MWM test measured cognitive ability. Through acquisition examinations, the average time to flee of the instructed rats decreased across all groups. When compared to control rats, AlCl_3_-induced animals had a considerably longer refractory period on the second, third, and fourth days of conditioning trials (*p* < 0.0001).

**Figure 3:**
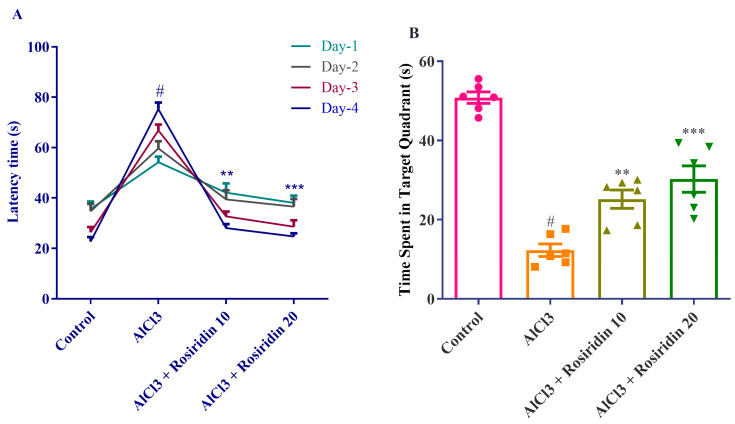
(**A**,**B**) Effects of rosirdin on MWM test. (**A**) Latency time by using two-way analysis of variance (ANOVA) by Bonferroni post-analytic test and (**B**) time spent in the target quadrant by using one-way ANOVA followed by Tukey’s test, respectively. Mean ± S.E.M. (*n* = 6). # *p* < 0.0001 vs. control; ** *p* < 0.001, *** *p* < 0.0001 vs. AlCl_3_-treated group.

AlCl_3_-treated rats’ latency times were considerably shortened by rosiridin administration, as demonstrated by a two-way ANOVA with a Bonferroni post hoc test. Beyond that, the post hoc test demonstrated that, on days 2, 3, and 4, rosiridin treatment (10 and 20 mg/kg) considerably lowered the delay period in contrast to rats given AlCl_3_ [F (3, 20) = 48.61, (*p* < 0.0001)] (Figure 3A). The rats’ ability to remember and recall the location of the obscure platform over the course of the four test days is assessed during the finalization phase.

Rosiridin treatment substantially affected the same (*p* < 0.0001), according to a one-way ANOVA that revealed AlCl_3_-treated rats expended a shorter period in the target region than the control rats. Furthermore, in contrast to AlCl_3_-treated rats, the post hoc test demonstrated that rosiridin (10 and 20 mg/kg) increased the amount of time invested in the desired zone [F (3, 80) = 3.831, (*P* = 0.0128)] (Figure 3B). These findings suggest that rosiridin effectively improved the cognitive impairment of AlCl_3_-treated rats.

#### 3.1.3. Open Field Test

**Figure 4:**
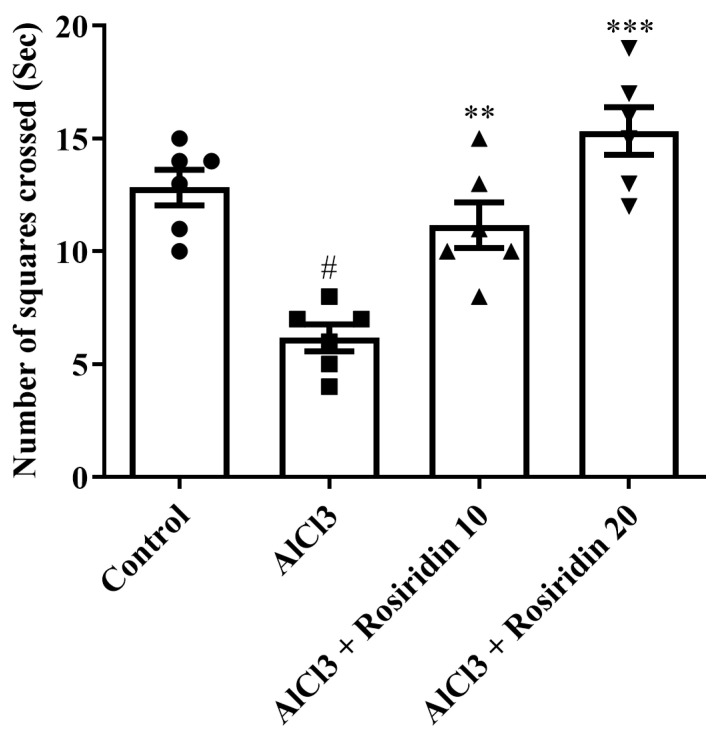
Effects of rosirdin on open field test by using one-way ANOVA followed by Tukey’s post hoc test, respectively. Mean ± S.E.M. (*n* = 6). # *p* < 0.0001 vs. control; ** *p* < 0.001, *** *p* < 0.0001 vs. AlCl_3_-treated group.

In the open field test, AlCl_3_-treated rats showed a significant reduction in locomotor activity, indicating impaired motor function. However, treatment with rosiridin (10 mg/kg and 20 mg/kg) improved locomotor activity in AlCl_3_-treated rats, as evidenced by increased total distance traveled and higher frequency of crossings in the open field (Figure 4). These findings suggest that rosiridin restored the motor function of AlCl_3_-treated rats.

### 3.2. Biochemical Analysis

#### 3.2.1. AChE and BuChE Determination

**Figure 5:**
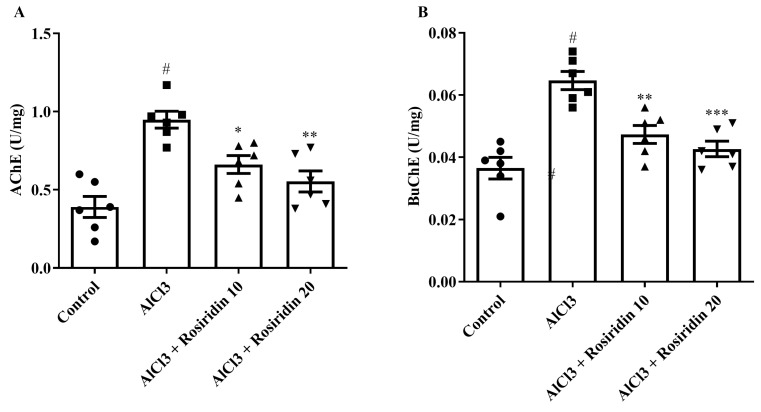
(**A**,**B**): Effect of rosiridin on (**A**) AChE and (**B**) BuChE by using one-way ANOVA followed by Tukey’s post hoc test, respectively. Mean ± S.E.M. (*n* = 6). # *p* < 0.0001 vs. control; * *p* < 0.05, ** *p* < 0.001, *** *p* < 0.0001 vs. AlCl_3_-treated group.

AChE and BuChE levels were used to evaluate cholinergic dysfunctions. The AlCl_3_-treated group exhibited statistically higher AChE and BuChE levels (*p* < 0.0001) compared to control rats. Rosiridin (10 and 20 mg/kg) significantly lowered AChE levels [F (3, 20) = 14.51, (*p* < 0.0001)] and BuChE levels [F (3, 20) = 16.75, (*p* < 0.0001)] compared to AlCl_3_-treated animals (Figure 5A,B). These findings suggest that the treatment of both doses of rosirdin (10–20 mg/kg) inhibited both AChE and BuChE in AlCl_3_-induced rats.

#### 3.2.2. Estimation of Antioxidant Parameters

**Figure 6:**
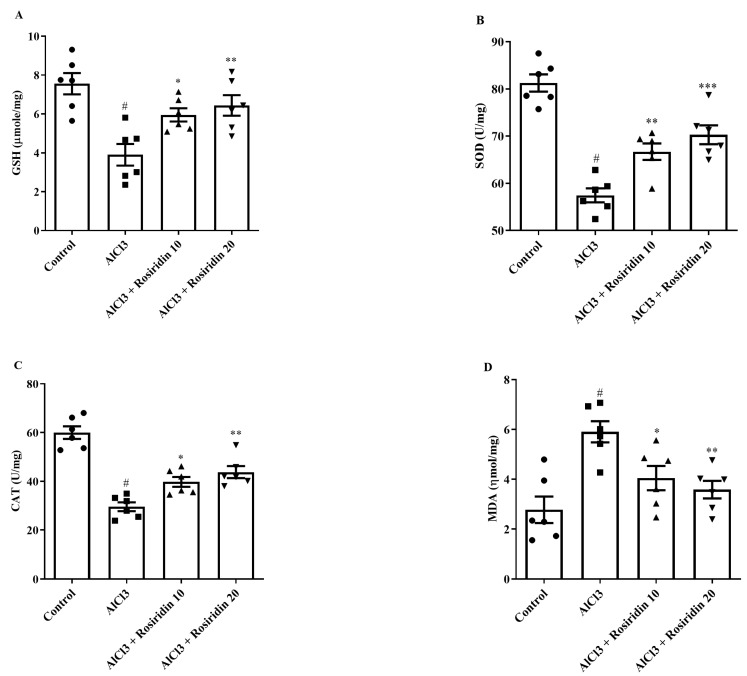
(**A**–**D**): effect of rosiridin on (**A**) GSH, (**B**) SOD, (**C**) CAT, and (**D**) MDA by using one-way ANOVA followed by Tukey’s post hoc test, respectively. Mean ± S.E.M. (*n* = 6). # *p* < 0.0001 vs. control; * *p* < 0.05, ** *p* < 0.001, *** *p* < 0.0001 vs. AlCl_3_-treated group.

Biological indicators were used to assess antioxidant enzyme activity. Compared to the control group, the GSH, SOD, and CAT levels were significantly lowered in the AlCl_3_-treated rat group. Treatment with both doses of rosiridin significantly elevated the GSH [F (3, 20) = 14.12, (*p* < 0.0001), SOD [F (3, 20) = 36.76, (*p* < 0.0001)], and CAT level [F (3, 20) = 27.49, (*p* < 0.0001)] compared to the AlCl_3_-treated group (Figure 6A–C).

Furthermore, the MDA level significantly increased in the AlCl_3_-treated group as compared to the control rats. Rosiridin at doses of 10 and 20 mg/kg significantly reduced MDA levels [F (3, 20) = 8.571, (*p* = 0.0007)] compared to AlCl_3_-treated rats (Figure 6D). Findings of this study revealed that rosiridin treatment at both doses significantly restores antioxidant enzyme activity impaired by AlCl_3_-treated rats.

#### 3.2.3. Estimation of Dopamine (DA), Serotonin (5-HT), and Ach

**Figure 7:**
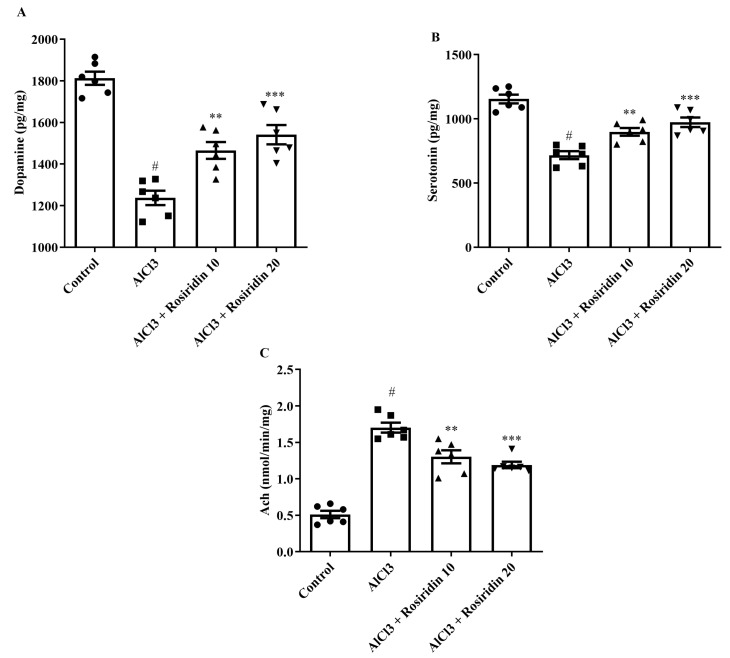
(**A**–**C**): Effect of rosiridin on (**A**) DA, (**B**) 5-HT, and (**C**) Ach by using one-way ANOVA followed by Tukey’s post hoc test, respectively. Mean ± S.E.M. (*n* = 6). # *p* < 0.0001 vs. control; ** *p* < 0.001, *** *p* < 0.0001 vs. AlCl_3_-treated group.

Neurotransmitter levels have been linked with learning and memory. Neurotransmitter levels evaluated the association between neurotransmitter levels and cognitive decline. AlCl_3_-treated rats exhibited statistically increased Ach and decreased DA and 5-HT levels compared to normal control rats (*p* < 0.0001). Rosiridin (10 and 20 mg/kg) treatment restored DA [F (3, 20) = 37.52, (*p* < 0.0001)], 5-HT [F (3, 20) = 29.65, (*p* < 0.0001)], and Ach [F (3, 20) = 57.54, (*p* < 0.0001)] levels as compared to AlCl_3_-treated rats (Figure 7A–C). The findings suggest that rosiridin treatment effectively mitigates AlCl_3_-induced alterations in DA, Ach, and 5-HT levels, thereby counteracting cognitive decline in rats.

#### 3.2.4. NO Content

**Figure 8:**
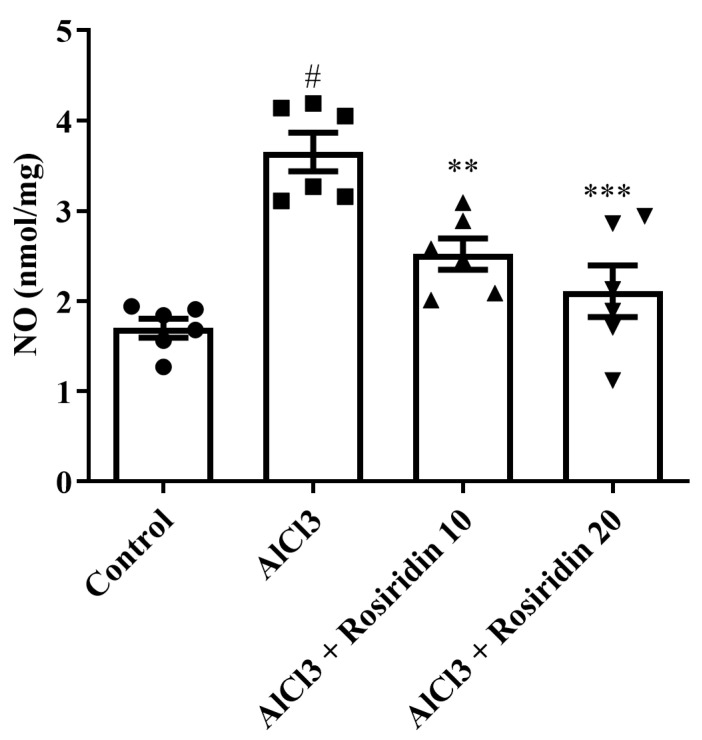
Effect of rosiridin on NO level by using one-way ANOVA followed by Tukey’s post hoc test, respectively. Mean ± S.E.M. (*n* = 6). # *p* < 0.0001 vs. control; ** *p* < 0.001, *** *p* < 0.0001 vs. AlCl_3_-treated group.

NO is a neurotransmitter that can induce cognitive behavior in the brain. The levels of NO in the rats were markedly increased in the AlCl_3_-treated group compared to the group not treated with AlCl_3_. In contrast, rosiridin at both doses significantly reduced NO levels [F (3, 20) = 16.83, (*p* < 0.0001)] compared to AlCl_3_-treated rats (Figure 8). The findings suggest that rosiridin treatment effectively alleviates AlCl_3_-induced alterations in NO levels, thereby counteracting cognitive decline in rats.

#### 3.2.5. Estimation of Neuroinflammatory Mediators

**Figure 9:**
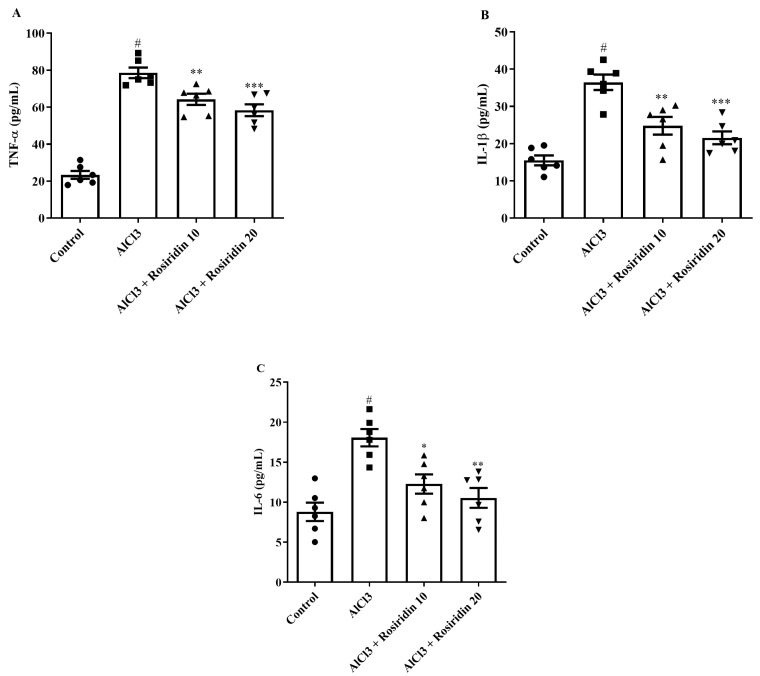
(**A**–**C**): Effect of rosiridin on (**A**) TNF-α, (**B**) IL-1β, and (**C**) IL-6 by using one-way ANOVA followed by Tukey’s post hoc test, respectively. Mean ± S.E.M. (*n* = 6). # *p* < 0.0001 vs. control; * *p* < 0.05, ** *p* < 0.001, *** *p* < 0.0001 vs. AlCl_3_-treated group.

Neuroinflammatory cytokines were used to evaluate anti-neuroinflammatory activity. AlCl_3_-treated rats exhibited statistically increased IL-1β, IL-6, and TNF-α levels compared to control rats. When rosiridin (10 and 20 mg/kg) was administered, the amount of IL-1β [F (3, 20) = 21.10, (*p* < 0.0001)], IL-6 [F (3, 20) = 11.78, (*p* < 0.0001)], and TNF-α [F (3, 20) = 68.16, (*p* < 0.0001)] reduced significantly in AlCl_3_-treated rats (Figure 9A–C). The findings suggest that rosiridin may inhibit neuroinflammatory cytokines in AlCl_3_-treated rats experiencing a neuroinflammatory response.

#### 3.2.6. Effect of Rosiridin on the BDNF, PI3K, pAkt, and NF-κB Levels

**Figure 10:**
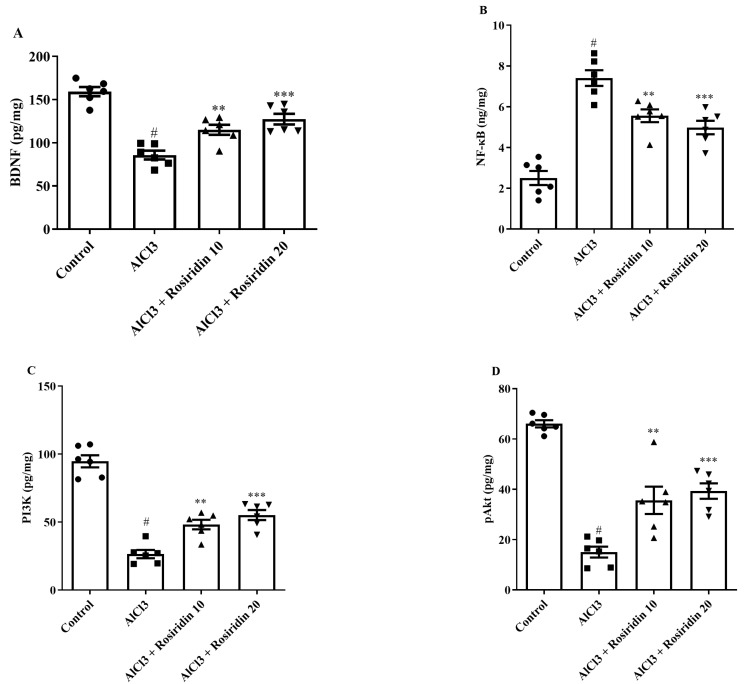
(**A**–**D**): Effect of rosiridin on (**A**) BDNF, (**B**) NF-ᴋB, (**C**) PI3K, and (**D**) pAkt by using one-way ANOVA followed by Tukey’s post hoc test, respectively. Mean ± S.E.M. (*n* = 6). # *p* < 0.0001 vs. control; ** *p* < 0.001, *** *p* < 0.0001 vs. AlCl_3_-treated group.

BDNF, PI3K, pAkt, and NF-κB levels were used to evaluate neuroprotective mechanisms, including neurotrophic support, cell survival signaling, and the regulation of inflammatory pathways in the brain. Figure 10A–D shows the results of measuring the levels of BDNF, PI3K, pAkt, and NF-κB in the cerebral regions of the treated and control animals. AlCl_3_-treated rats demonstrated an elevation in NF-κB and downregulation of the BDNF, PI3K, and pAkt levels compared to the control group. Treatment with rosiridin (10 and 20 mg/kg) resulted in downregulation of the NF-κB [F (3, 20) = 34.62, *p* < 0.0001] levels and upregulation of the BDNF [F (3, 20) = 29.38, (*p* < 0.0001)], PI3K [F (3, 20) = 58.82, (*p* < 0.0001)] and pAkt [F (3, 20) = 38.79, (*p* < 0.0001)] levels compared to AlCl_3_-treated rats (Figure 10A–D). Rosiridin’s effects include enhancing neuroprotective mechanisms by modulating BDNF, PI3K, pAkt, and NF-κB levels.

## 4. Discussion

The current investigation considered the neuroprotective impact of rosiridin on AlCl_3_-produced memory deterioration in rats. Metal exposure is harmful and dangerous, and it may cause neurodegeneration due to oxidative stress. AlCl_3_ is a necessary metal, but too much of it can be hazardous to the brain. Al alters cholinergic transmission and lowers the amount of Ach in the brain [59,60]. Earlier research demonstrated that long-term AlCl_3_ stimulation impairs memory and cognition because it alters the neurons [61,62,63]. The outcome of the current investigation illustrates that rosiridin has antioxidant action by reducing inflammatory cytokines, metabolic markers, and improving cognitive function in rats exposed to AlCl_3_ [64,65,66].

The results of the Y-maze and MWM tests, which assess rats’ memory and cognitive abilities, changed after exposure to AlCl_3_ [67,68]. Our study found that AlCl_3_ substantially (*p* < 0.05) lowered arm entry and spontaneous alteration (Y-maze), and significantly increased escape latency (MWM). Rosiridin considerably improved memory strength [39]. The Y-maze and MWM tests demonstrated that both doses of rosiridin substantially (*p* < 0.0001) exacerbated the cognitive deficiency induced by AlCl_3_. Furthermore, open field test indicated that rosiridin significantly increased the exploratory ability in a novel environment. The findings suggest that rosiridin mitigates memory impairment and partially restores motor function, ruling out the possibility that the improvements observed in the Y-maze and Morris Water Maze tests were solely due to changes in locomotor activity. The findings of the study agreed with earlier reports that behavioral tests showed that rosiridin enhanced the rats’ ability to learn and coordinate their movements after exposure to AlCl_3_ [69].

According to several studies [50,58], AD is associated with dysfunctions of the cholinergic system that involve AChE and BuChE enzymes. AChE is an essential A chemical within the cerebral cortex that maintains the integrity of the cholinergic neuronal membrane and breaks down Ach. By changing the cholinergic route and transmission, Al exposure damages the cholinergic system [70,71]. Similarly to past studies, AlCl_3_ significantly increased AChE and BuChE levels in the current investigation. The rosiridin-treated groups exhibited significantly reduced AChE and BuChE, improving brain cognitive abilities. The results of this study are in accordance with a previous study [72].

Reactive oxygen species, produced by oxidative stress, are the primary factor in neuronal dysfunction, inflammation, and, ultimately, cell death, which results in neurodevelopmental diseases. The body’s natural antioxidant production is the initial layer of defense from oxidative stress. Antioxidants reduce the creation of free radicals, which reduces oxidative stress [73]. To counteract oxidative stress, the enzyme CAT converts hydrogen peroxide into water and oxygen, while SOD protects against oxidative damage by catalyzing the conversion of superoxide anions into less harmful molecules [74]. Previous investigations show that the current investigation demonstrated that AlCl_3_ lowered GSH, SOD, and CAT levels in experimental animals, accelerated oxidant harm [15,75]. Both doses of rosiridin showed a considerable increase in the antioxidative parameters. Moreover, AlCl_3_ increased the MDA levels and NO in the rat brain, as reported in other investigations [76]. Rosiridin helps restore NO levels, indicating its protective role against oxido-nitrosative stress. Thus, the reported findings demonstrate the antioxidant capacity of rosiridin and its ability to mitigate oxidative damage resulting from AlCl_3_-induced neurotoxicity in rats, which agreed with the reported study [41].

It has been proposed that neurotransmitters perform an essential part in neuroregulation, which leads to memory loss and social dysfunction in AD [68]. Intellectual difficulties and impairment in AD are strongly connected by neuron degeneration and the dysfunction of parasympathetic neural cells, resulting in the main focus of the “cholinergic hypothesis” being overall neurotransmitters [68]. The mesencephalon generates DA, which diffuses to various brain regions involving the hippocampus, cortex, and basal ganglia. Dopaminergic problems have been established as an assisting element in the pathophysiology of memory decline reported by persons with indications of AD [68]. The current study demonstrates that the AlCl_3_-administered AD animals were found to lower the amount of DA and 5-HT while enhancing the Ach levels in the brains of rats. However, rosiridin restored the DA, Ach, and 5-HT levels. The results of this study concord with a previous study [40].

The body’s defensive mechanism, which detects inflamed tissues, comprises inflammatory mediators. In cases of neuroinflammation, the brain’s NF-κB activates inflammatory mediators like IL-6, IL-1β, and TNF-α to start the modulation of inflammation process [77]. According to the study’s findings, rats exposed to AlCl_3_ had higher levels of the cytokines TNF-α, IL-1 β, and IL-6, which is consistent with earlier findings [78,79]. Meanwhile, rosiridin dramatically decreased these levels in both groups. The results indicate rosiridin’s potential to reduce inflammation and protect rats against AlCl_3_-induced neurotoxicity which helps maintain NO at physiological levels, thereby protecting neurons and improving memory function, which was in accord with a prior work [40].

Inflammation is the initial AD event that causes aberrant occurrences. The nuclear factor NF-κB activates inflammation-related genes. Injury in neurodegenerative illnesses like Alzheimer’s activates NF-κB, particularly in specific brain areas. After neuroinflammation, IκBα degradation leads to the nuclear distribution of NF-kβ. Consequently, this activation initiates an inflammatory response. Conversely, rosiridin shows an important inhibitory outcome on the AlCl_3_-administered rats with elevated levels of NF-κB, which is identified as an inflammatory marker [68].

It has previously been reported that low BDNF levels in AD are considered a marker of neurodegeneration and disease progression [80]. The treatment with AlCl_3_ decreased BDNF expression. The study found that BDNF causes synapse loss due to the harmful effects of Aβ. Rosiridin restored BDNF levels in AlCl_3_-induced rats, which was also confirmed by a previous study in a rat model [68]. The PI3K–Akt signaling pathway is a complex cellular cascade engaged in different biological activities, such as transmitting signals and cell growth, apoptosis, and metabolism. Several studies have shown that AD represses the PI3K–Akt axis, restoring its activity and ameliorating Aβ-induced neurotoxicity. As previously reported, AlCl_3_ suppressed PI3k/pAkt levels and rosiridin reversed AlCl_3_’s inhibitory effect, which agreed with another study [58].

Previous research has indicated that the pathophysiology of AD, especially the formation of amyloid plaques from Aβ synthesis, is significantly influenced by MAO activation. Plant-derived natural products such as rosiridin have the potential to inhibit MAO-A and MAO-B [40]. Compared to traditional MAO inhibitors, rosiridin may offer advantages such as higher patient acceptability, multi-targeted therapeutic effects, fewer side effects, and broader potential applications in treating neurological conditions like dementia and cognitive impairment. These factors may make rosiridin a safer and more effective alternative in future studies.

**Figure 11:**
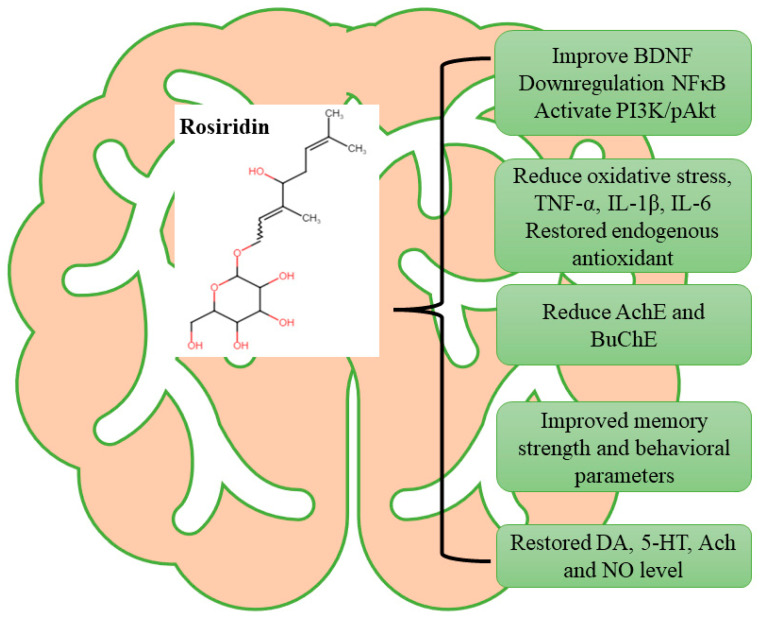
Hypothetical mechanism of rosiridin.

This study highlights rosiridin’s significant effects on restoring behavioral parameters and various biochemical markers related to oxidative stress, neurotransmitter balance, neuroinflammatory cytokines, and PI3K/pAkt levels in rats (Figure 11). This pioneering approach provides a new perspective on rosiridin’s therapeutic benefits for memory impairment and its broader application in neurodegenerative disorders.

We observed that the higher dose (20 mg/kg) of rosiridin resulted in more pronounced improvements in behavioral outcomes and biochemical parameters than the 10 mg/kg dose. The study was limited by its short duration, minimal animal use, the inclusion of only male rats the analysis of cerebral tissues without examining specific subregions in the cortex or hippocampus, Western blotting, and immunochemistry.

## 5. Conclusions

The present research demonstrates that rosiridin maintained the behavioral and biochemical abnormalities that AlCl_3_ causes in rats. The antioxidant and anti-inflammatory properties of rosiridin suggest that it may have positive effects against AlCl_3_-treated rats. More research is needed to discover whether rosiridin can aid patients with neurodegenerative illnesses.

## Data Availability

All data presented in this study are included in the article.
